# Single-Cell Transcriptomics Applied in Plants

**DOI:** 10.3390/cells13181561

**Published:** 2024-09-17

**Authors:** Yanyan Sun, Jian Sun, Chunjing Lin, Jingyong Zhang, Hao Yan, Zheyun Guan, Chunbao Zhang

**Affiliations:** 1 Soybean Research Institute, Jilin Academy of Agricultural Sciences, Changchun 130033, China; sunyy@cjaas.com (Y.S.); lincj@cjaas.com (C.L.); zhangjy@cjaas.com (J.Z.); yanhaoonline@163.com (H.Y.); zyguan@cjaas.com (Z.G.); 2 Institute of Agricultural Quality Standard and Testing Technology, Jilin Academy of Agricultural Sciences, Changchun 130033, China; 15591888869@163.com; 3 Key Laboratory of Hybrid Soybean Breeding, Ministry of Agriculture and Rural Affairs, Changchun 130033, China

**Keywords:** single-cell RNA sequencing, model plants, crops, wood, databases

## Abstract

Single-cell RNA sequencing (scRNA-seq) is a high-tech method for characterizing the expression patterns of heterogeneous cells in the same tissue and has changed our evaluation of biological systems by increasing the number of individual cells analyzed. However, the full potential of scRNA-seq, particularly in plant science, has not yet been elucidated. To explore the utilization of scRNA-seq technology in plants, we firstly conducted a comprehensive review of significant scRNA-seq findings in the past few years. Secondly, we introduced the research and applications of scRNA-seq technology to plant tissues in recent years, primarily focusing on model plants, crops, and wood. We then offered five databases that could facilitate the identification of distinct expression marker genes for various cell types. Finally, we analyzed the potential problems, challenges, and directions for applying scRNA-seq in plants, with the aim of providing a theoretical foundation for the better use of this technique in future plant research.

## 1. Introduction

Plants are immobile; therefore, they need to carry out frequent and significant transcriptional programs to cope with the challenges brought about by the environment and complete the process of development. Different cell types, including various developmental stages of the same cell type, have different biological functions during plant development and environmental adaptation. Additionally, the levels of transcriptional activity across genes are distributed across various cell types. Therefore, plant cell heterogeneity should not be ignored when exploring the transcriptional responses to various internal and external signals. Nevertheless, traditional RNA-Seq primarily uses parts of the tissues, organs, or even entire organisms as samples. The results of traditional RNA-Seq are the overall average expression of hundreds or even thousands of cells, which can only capture the expression levels of highly expressed genes. As a result, genes with low expression levels were always covered, and cell heterogeneity was ignored.

For the past few years, the fast growth of single-cell RNA sequencing (scRNA-seq) has compensated for the shortcomings of traditional RNA-seq and addressed the challenges posed by cellular diversity and limited sample accessibility. These approaches allow scientists to explore plant tissues with a high resolution and throughput [1]. Phytobiology has fully adopted scRNA-seq and rapidly expands the available technologies and applications [2]. scRNA-seq uses a distinct cell as the detection unit, thus enabling the obtainment of the transcriptome information of a single cell, which is meaningful for revealing the plant development process, studying evolutionary relationships between species, and identifying new cell types.

However, because the preparation of a single-cell suspension of a plant requires the digestion of the cell wall, previous research on single-cell suspensions have primarily focused on model plants, including *Arabidopsis thaliana* (*A. thaliana*) [3,4,5,6,7,8] and rice (*Oryza sativa*) [9,10,11,12]. Furthermore, protoplast-based protocols for scRNA-seq in plants have certain limitations. First, single-cell dissociation induces a strong transcriptional stress response. Second, different samples exhibit different dissociation deviations. Third, it is difficult to prepare single-cell suspensions for partial plant samples [13]. To address these issues, certain investigations have opted for single-nucleus RNA sequencing (snRNA-seq) as a substitute for scRNA-seq. Nowadays, with continuous improvements in technology, scRNA-seq and snRNA-seq technologies are increasingly being applied to other plants, such as soybean (*Glycine max*) [14,15,16,17], *M. truncatula* (*Medicago truncatula*) [18,19,20,21,22], peanuts (*Arachis hypogaea*) [23,24,25,26,27], and others. It is apparent that revolutions in plant research are now possible with the advent of scRNA-seq and snRNA-seq, providing unparalleled prospects.

## 2. Milestones in scRNA-Seq Development

Single-cell sequencing is an innovative technique that enables the analysis of genomic, transcriptomic, and epigenomic data at a single-cell resolution using high-throughput sequencing methods. The single-cell sequencing technology can analyze the specificity of important rare cells, detect the diversity among cells in tissues, and solve other problems that cannot be solved through traditional sequencing technology. Moreover, its applications encompass the recognition of cellular categories, the unveiling of unique functionalities, and the demonstration of changes in specific cells. Currently, it finds extensive utilization across various domains, including neurobiology, tumor research, clinical diagnosis, microbiology, and other fields of plant development [28,29,30,31]. It mainly includes single-cell RNA sequencing (scRNA-seq), single-cell genome sequencing (scDNA-seq), single-cell genome-wide bisulfite sequencing (scWGBS), single-cell assays for transposase-accessible chromatin with high-throughput sequencing (scATAC-seq), single-cell adaptive immune receptor sequencing (scVDJ-seq), and single-cell Hi-C (scHi-C). The aforementioned techniques, including scDNA-seq, scWGBS, and scVDJ-seq, have been extensively employed in the diagnosis and treatment of human diseases [32,33,34]. ScATAC-seq, scHi-C, and scRNA-seq have been utilized in *A. thaliana* and rice, while there has been a substantial increase in publications focusing on scRNA-seq in recent years [1,35,36].

### 2.1. Low-Throughput Cell Sorting Period

In the last twenty years, progressions in next-generation sequencing (NGS) and microfluidic technology have played a crucial role in accurately mapping the transcriptome at a cellular level [2]. Tang et al. first described a novel method for analyzing gene expression profiles at single-cell level using digital technology in mice. This approach enabled them to identify a significantly higher number of expressed genes compared to traditional microarray techniques and discover numerous splice junctions that were previously unknown (1753, to be precise). Additionally, they observed that a considerable proportion of genes with multiple known transcript isoforms exhibited the co-expression of at least two isoforms within the same cell sample, highlighting the intricate nature of transcript variants across an entire genome within individual cells [37]. Subsequently, scRNA-seq has undergone continuous advancements and found extensive applications in various scientific investigations.

**Figure 1:**
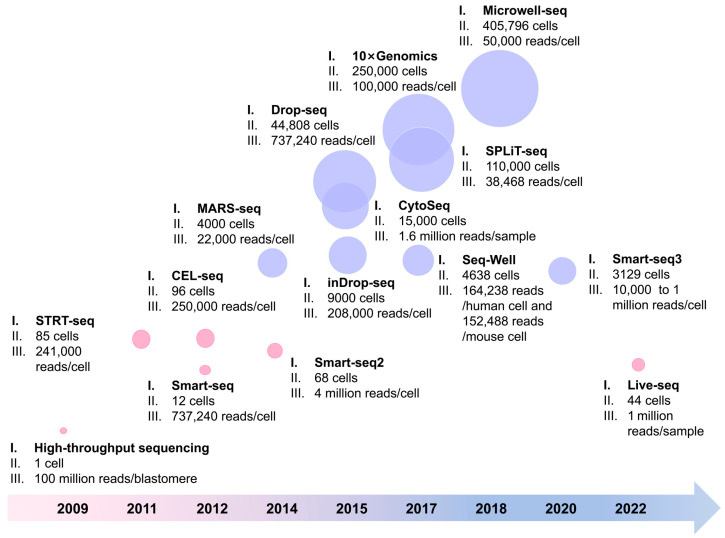
The main technology used in single-cell RNA-sequencing. I, technique; II, cell numbers; III, sequencing depth. Pink circles represent low-throughput sequencing technology; purple circles represent high-throughput sequencing technology. The size of the circle indicates the number of cells.

In 2011, Islam et al. established STRT-seq, a single-cell-tagged reverse transcription sequencing technique that utilizes an Illumina sequencing platform [38]. This approach eliminates the need for marker genes and enables the differentiation of cell types by analyzing their unique gene expression profiles. However, this method is unable to amplify the entire transcript, resulting in the inability to partition the variable splicing events in the 5′ and 3′ UTR regions. Ramsköld et al. established the Smart-seq technique for RNA transcript sequencing, focusing on the 5′ end of the sequence, and this method was performed on mouse oocytes [39]. Smart-seq can significantly improve the full-length coverage of transcripts larger than 1 kb and enable a more detailed analysis of variable splicing events. In the same year, Hashimshony et al. established cell expression by linear amplification and sequencing (CEL-seq) [40]. This technique uses linear mRNA amplification for the single-cell sequencing of mammalian cells and nematode embryonic blastomeres with high specificity and barcode efficiency. Compared to STRT-seq, CEL-seq exhibits superior stability, sensitivity, and reproducibility, as well as reduced technical noise, except for the bias in the 3′ UTR region. Picelli et al. optimized Smart-seq and established Smart-seq2 [41]. The improvements made to Smart-seq2 encompassed optimizing the reverse transcription, template replacement, and pre-amplification techniques, ultimately leading to an increase in both the quantity and length of the cDNA libraries produced from individual cells. In addition, Smart-seq2 optimizes the detection range, coverage, bias, and accuracy, and even reduces the sequencing costs. However, it needs high-quality RNA, as the presence of degraded RNA could potentially lead to the omission of mRNA information within the 5′ UTR region. Moreover, most lncRNA information is lost, and the efficiency of database construction for transcript data over 4000 bp is still low. Chen et al. developed Live-seq, a novel method for profiling the transcriptome of individual cells while maintaining their viability during RNA extraction [42]. This innovative approach utilizes fluidic force microscopy (FluidFM), enabling the correlation between a cell’s baseline gene expression profile and its subsequent molecular or phenotypic behavior. However, Live-seq still has disadvantages; for example, it is a low-throughput method that is not yet available in vivo. Furthermore, in highly polarized cells with an uneven mRNA distribution (such as nerve cells), Live-seq may not embody the whole-cell transcriptome. During this stage, the quantity of single cells analyzed in each research for single-cell transcriptomic experiments has increased from 10 to 100, resulting in an improved throughput [43] (Figure 1).

### 2.2. High-Throughput Cell Sorting Period

With the development and improvement of microporous, microfluidic, microdroplet, and in situ barcode technologies, scRNA-seq has entered a new era of high throughput and automation. Jaitin et al. introduced a high-throughput RNA single-cell sequencing framework called MARS-seq, which utilizes automation and parallel processing [44]. The technique is specifically designed for the in vivo sampling of numerous cells through multiplexed RNA-seq, while ensuring precise control over amplification biases and labeling errors. By utilizing fluorescence-activated cell sorting (FACS), a single cell was placed in a 384-well plate for sequencing and subsequent automated processing. The majority of the processing involved pooled and labeled material, resulting in a remarkable enhancement in both the throughput and the reproducibility.

Subsequently, based on droplet microfluidic technology, Macosko et al. developed droplet-based RNA-seq (Drop-seq), which introduces single cells and barcode beads into nanoscale oil-coated water droplets, lyses them, and captures mRNA in a water-in-oil drop [45]. This method can sequence tens of thousands of single cells; however, it is expensive. This is the first technique applied to plant scRNA-seq [3]. Klein et al. devised a high-throughput droplet microfluidic technique called inDrop (indexing droplets) RNA sequencing, which enables the barcoding of RNA from numerous individual cells for subsequent examination using next-generation sequencing [46]. This approach exhibits an unexpectedly minimal level of noise and can be easily adapted for other sequencing-based assays. One notable technical limitation is the mRNA capture efficiency, which stands at approximately 7%. Although this efficiency surpasses that of several previously published methods, it remains insufficient for reliably detecting genes exhibiting transcript abundances below 20–50 transcripts per cell. Another drawback pertains to the random barcoding strategy employed by this method, preventing the association of specific cell identities with respective barcodes. Fan et al. introduced a scalable method that allows for the routine profiling of digital gene expression in thousands of single cells across any number of genes, without relying on robotics or automation [47]. This technique, known as CytoSeq, utilizes a recursive Poisson approach. Currently, this method can simultaneously analyze several thousand cells and has the potential to scale up effortlessly to tens or hundreds of thousands of cells.

The 10× chromium single-cell gene expression solution is a commercial high-throughput sequencing technology based on the microfluidic technology introduced by 10× Genomics in 2016 [48]. This is an important milestone in scRNA-seq, which allows for the genome-wide detection and the simultaneous expression profiling of thousands of cells. The highlight of this technology is that the tissue cell population is divided into nanoscale gel microbeads (GEMs), which are combined to form oil–water droplets, and then the cells are broken up to obtain mRNA for reverse transcription. cDNAs of the same cell type have a common cell barcode, and an analysis of the cell group can be carried out. The 10× Genomics platform covers a wider range of cells, and it is easier to detect rare cell types in the sequencing process. However, the platform has certain limitations. For one thing, only 3′ end transcript information can be obtained, and a significant quantity of living cells are required (approximately 105–106), which is more restrictive for rare samples. In addition, there are strict requirements for cell extraction [49].

Gierahn et al. presented Seq-Well, an affordable and portable system designed for the high-throughput analysis of individual cell RNA sequences [50]. By employing barcoded mRNA capture beads and sealing them with single cells within sub-nanoliter wells through a semi-permeable membrane, this method enables the effective extraction of cellular content and transcription data acquisition. The versatility of this robust approach facilitates the application of single-cell transcriptomics across various types of cellular suspensions that possess either known reference genomes or transcriptomes. In the same year, Rosenberg et al. developed split-pool, ligation-based transcriptome sequencing (SPLiT-seq) technology [51]. This method does not require the separation of a single cell. Depending on the gaps in the cell, cells fixed with formaldehyde can be used as reaction chambers, and specific barcodes in single cells can be used to label RNA. This reduces the influence of endogenous gene expression during cell processing, improves sequencing throughput, and reduces costs.

In 2018, Microwell-seq technology was developed by Han et al., who used nanopores constructed by agarose to capture single cells and then used magnetic beads to capture mRNA [52]. Using this method, Han et al. conducted an analysis on over 400,000 individual cells encompassing the major organs of mice. They employed a cost-effective and high-throughput scRNA-seq platform that utilizes affordable and uncomplicated devices. By employing a silicon wafer containing approximately 100,000 microwells, they successfully produced multiple sets of polydimethylsiloxane (PDMS) micropillar arrays. These arrays were reusable and facilitated the creation of numerous agarose microwell arrays on multiple occasions. The efficient collection was facilitated by the magnetic properties of the barcoded beads, while any remaining beads outside the microwells were recycled for further use. The estimated cost for generating sequencing libraries for each cell type was less than 0.02 USD.

In 2020, Hagemann-Jensen et al. established a new method called Smart-seq3, which integrates extensive transcriptome coverage with a distinctive RNA counting strategy using molecular identifiers at the 5′ end [53]. This innovative technique enables the virtual reconstruction of numerous RNA molecules within each cell. Approximately 60% of these identified and reconstructed molecules can be directly linked to their respective allelic source, while approximately 30–50% can be attributed to specific isoform. Compared to its predecessor Smart-seq2, Smart-seq3 exhibits significantly enhanced sensitivity and is capable of detecting thousands of transcripts in individual cells. Over time, scRNA-Seq technology has made remarkable strides from initially capturing only a limited number of single cells to now encompassing tens of thousands for sequencing purposes (Figure 1).

## 3. scRNA-Seq Technologies Used in Plant Research

**Figure 2:**
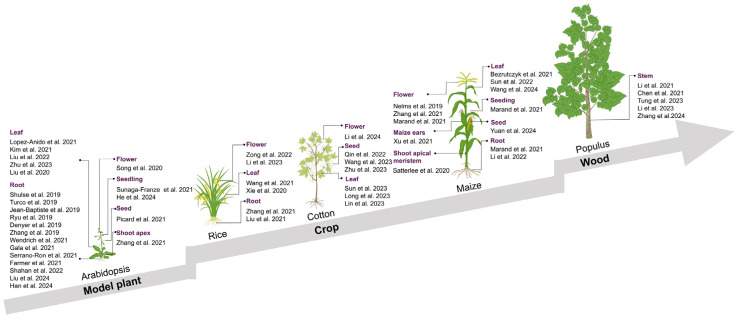
Application of single-cell RNA-sequencing in major model plants, crops, and wood [3,4,5,6,7,8,9,10,11,12,55,56,57,58,59,60,61,62,63,64,65,66,67,68,69,70,71,72,73,74,75,76,77,78,79,80,81,82,86,87,88,89,90,91,92,101,102,103,104,105].

Creating detailed maps of organs at the cellular level can enhance our comprehension of plant-specific cell types and diverse biological inquiries, including the developmental paths of cells, their reactions to biotic and abiotic stressors, and the variations and commonalities in cell types among diverse species, among other aspects [6,7,12,54]. However, in plants, the progress in scRNA-seq has been limited due to the hindrance posed by plant cell walls, which impede the ability to isolate and gather single cells. The advent of cost-effective and high-throughput cell processing techniques has paved the way for droplet-based single-cell methodologies to dominate the field of plant single-cell transcriptomics. These methods, such as Drop-seq and 10× Genomics, have found extensive applications across various domains including model plants, crops, and even wood (Table 1, Figure 2).

**Table 1:** scRNA-Seq technologies used in mainly plant research.

Species	Samples	Cell/Nucleus Numbers	Platforms	References
*Arabidopsis thaliana*	Root	12,198	Drop-seq	[3]
	Root	7522	10× Genomics	[4]
	Root	2800	Drop-seq	[5]
	Root	4727	10× Genomics	[6]
	Root	3121	10× Genomics	[7]
	Root	7695	10× Genomics	[8]
	Root	282	Smart-Seq2	[55]
	Root	6658	10× Genomics	[56]
	Root	110,427	10× Genomics	[57]
	Root	5145	10× Genomics	[58]
	Root	10,548	10× Genomics	[59]
	Root	16,670	10× Genomics	[60]
	Root	149 gene co-expression modules	Publicly available datasets	[61]
	Cotyledon	14,117	10× Genomics	[62]
	Cotyledon	12,844	10× Genomics	[63]
	Leaf	11,895	10× Genomics	[64]
	Leaf	13,000	10× Genomics	[65]
	Leaf	5230	10× Genomics	[66]
	Shoot apex	36,643	10× Genomics	[54]
	Egg, central, and synergid cells	Eight cells for each cell type	NextSeq 500 platform	[67]
	Seed	1437	Smart-Seq2	[68]
	Seedling	3348	NextSeq 500 platform	[69]
	Seedling	186,030	10× Genomics	[70]
*Zea mays*	Anther	144	CEL-seq2	[71]
	Shoot apical meristem	327	CEL-seq2	[72]
	Developing maize ears	12,525	10× Genomics	[73]
	Meiocytes (tetrad stage) and early mononucleate microspores	48	Illumina MiSeq platform	[74]
	Seedling, root tips, crown root, inflorescence, axillary bud, *A. thaliana* whole roots	15,515	10× Genomics	[75]
	Root	10,000	10× Genomics	[76]
	Abaxial epidermis	33,098	10× Genomics	[77]
	Endosperm	17,022	10× Genomics	[78]
	Ligular region of leaf	7049	10× Genomics	[79]
	Leaf	3763 and 3242 cells from the first and second scRNA-seq experiment	10× Genomics	[80]
*Oryza sativa*	Root	27,469	10× Genomics	[12]
	Root	23,532	10× Genomics	[9]
	Leaf and root	237,431	10× Genomics	[10]
	Leaf	12,891 and 13,639 cells were captured in the control and TOE	10× Genomics	[11]
	Inflorescence	37,571	10× Genomics	[81]
	Pistil	8173	10× Genomics	[82]
*Triticum aestivum*	Root	15,000	10× Genomics	[83]
*Manihot esculenta*	Tuberous root	14,566	10× Genomics	[84]
*Pisum sativum*	Shoot apices	14,493	10× Genomics	[85]
*Gossypium*	Ovule	14,535	10× Genomics	[86]
	Ovule	18,064 WT and 21,389 fl cells	10× Genomics	[87]
	Hypocotyl	30,357 cells from Jin668; 29,234 cells from TM-1	10× Genomics	[88]
	Cotyledon	9186	10× Genomics	[89]
	Cotyledon	13,495	10× Genomics	[90]
	True leaves	28,655	10× Genomics	[91]
	Bud	25,050	10× Genomics	[92]
*Brassica rapa*	Shoot and leaf	12,985 shoot cells and 17,245 leaf cells	10× Genomics	[93]
	Leaf	16,055	10× Genomics	[94]
*Solanum lycopersicum*	Shoot apex	13,377	10× Genomics	[95]
	Callus	28,036	10× Genomics	[96]
*Nicotiana attenuata*	Corolla limbs and throat cups	3756	10× Genomics	[97]
*Lotus japonicus*	Root	22,688	10× Genomics	[98]
	Root	25,024	10× Genomics	[99]
	Nodules	50~100	Smart-seq2	[100]
*Glycine max*	Nodules and roots	14,369 nuclei from root; 7830 nuclei from nodule	10× Genomics	[14]
	Nodules and roots	26,712 nuclei	10× Genomics	[15]
	Root	23,063 and 19,712 transcriptomes from the N and R groups	10× Genomics	[16]
	Hypocotyl, root, nodule, and four stages seeds	115,626 nuclei	10× Genomics	[17]
*Medicago truncatula*	Nodules	9756	10× Genomics	[18]
	Root	25,276	10× Genomics	[19]
	Root	15,854 inoculated and 12,521 mock-inoculated nuclei	10× Genomics	[20]
	Root	16,211	10× Genomics	[21]
	Root	16,890	10× Genomics	[22]
*Arachis hypogaea*	Leaf blade	6815	10× Genomics	[23]
	Leaf blade	24,988	10× Genomics	[24]
	Leaf	13,409 and 11,296 single cells from leaves grown under dark and light	10× Genomics	[25]
	Leaf	5930	10× Genomics	[27]
	Fruit	13,230, 20,030, and 16,636 cells from Aerpeg, Subpeg, and Exppod	10× Genomics	[26]
*Populus*	Stem-differentiating xylem	9798	10× Genomics	[101]
	Stem	3626 bark tissue cells and 3170 wood tissue cells	10× Genomics	[102]
	Stem-differentiating xylem	4705	10× Genomics	[103]
	Primary growth tissue and secondary growth tissue	7416 primary growth tissue cells and 11,769 secondary growth tissue cells	10× Genomics	[104]
	Xylem and phloem	10,516 and 9553 cells from WT and *myb31-7* mutant	10× Genomics	[105]

### 3.1. Research and Application in Model Plant Arabidopsis Thaliana

To date, several studies have developed gene expression maps of *Arabidopsis* for various tissues on a cellular level, including roots [3,4,5,6,7,8,55,56,57,58,59,60,61], leaves [62,63,64,65], blade vascular systems [66], vegetative shoot apexes [54], gametophytes [67], seeds [68], and even seedings [69,70].

#### 3.1.1. Research on Root Tissue

In 2019, Shulse et al. performed pioneering work on scRNA-seq in plant fields [3]. They conducted a high-throughput Drop-seq analysis on *Arabidopsis* roots, resulting in the generation of a comprehensive transcriptional dataset for over 12,000 cells representing various populations. This investigation unveiled specific genes that serve as markers for various types of cells encompassing all primary root tissues and developmental phases. Additionally, it examined alterations in cell frequency following the addition of sucrose and demonstrated the dynamic regulation of genes throughout the developmental process.

Subsequently, there has been an increase in the number of scRNA-seq studies conducted on *Arabidopsis* roots. Ryu et al. utilized 10× Genomics to acquire single-cell transcriptomes from over 10,000 protoplasts of *Arabidopsis* root cells [4]. By employing this technique, they successfully identified distinct subpopulations and rare cell types, like potentially dormant central cells. Moreover, they showcased the significance of comparative analyses between the scRNA-seq data obtained from wild-type and mutant roots in elucidating the functional role of genes at a cellular level. Shahan et al. developed an *Arabidopsis* root atlas which unveiled gradual alterations in gene expression during cell type differentiation and identified potential regulators influencing cell fate determination [57]. This comprehensive atlas not only facilitated the interpretation of smaller scRNA-seq datasets but also revealed novel phenotypes associated with developmental mutants. Denyer et al. produced an extensive gene expression atlas by mapping the individual cellular transcriptome of *Arabidopsis* root cells [6]. This atlas captured the spatial and temporal data pertaining to the primary cellular categories and revealed previously unknown regulators. By analyzing pseudotime data, the researchers observed a series of developmental progressions from stem cells to differentiated cells, accompanied by waves of transcription factor expression. Zhang et al. conducted a study on gene expression in numerous individual *Arabidopsis* root cells [8]. Their findings revealed significant heterogeneity among root cells, even within the same cell type. By employing pseudotime analyses, they were able to construct a continuous trajectory depicting the differentiation of these root cells. Additionally, they developed an internet-based server (http://wanglab.sippe.ac.cn/rootatlas/) (accessed on 25 July 2024) to facilitate easy access to and the utilization of the datasets generated during their research.

Cellular developmental trajectories have also been utilized for the examination of *Arabidopsis* lateral root development. In a recent study by Gala et al., they investigated the transcriptomes of initial lateral root primordia in *Arabidopsis* and identified numerous genes that were upregulated during this process [56]. A novel approach was devised to selectively inhibit the transcription of target genes in xylem pole pericycle cells, which serve as the origin for lateral roots. Additionally, distinct cell subpopulations within the pericycle and endodermal cell fields were found to exhibit responses to the initiation of lateral roots. Serrano-Ron et al. employed scRNA-seq to study the initial four stages of lateral root formation in *Arabidopsis* [55]. Their findings demonstrated that the development of lateral root primordium is well coordinated and involves the arrangement of cell lineages according to conserved sequences within three distinct developmental pathways. Additionally, they discovered an associated role of camalexin in one of the three developmental trajectories.

The utilization of scRNA-seq enables the investigation of plant cellular reactions towards biotic and abiotic pressures at a highly detailed level. Jean-Baptiste et al. employed 10× Genomics technology on *Arabidopsis* roots for capturing gene expressions from a total of 3121 root cells [7]. Through conducting a pseudotime analysis on various cellular lineages, they successfully identified numerous genes exhibiting specific expressions within distinct cell types along their developmental path—encompassing both previously recognized and newly discovered genetic elements. Furthermore, when subjecting entire seedlings to heat stress conditions, it was observed that while the dominance in gene expression across different cell types is attributed to canonical heat-shock genes’ response, subtle yet noteworthy variations were also discerned among these cellular populations in terms of other genetic components. Recently, Liu et al. conducted an analysis on *Arabidopsis* root tip nuclei, examining gene expression and chromatin accessibility [60]. Their aim was to reconstruct transcriptional regulatory networks (TRNs) specific to different cell types involved in the development of root tips under osmotic stress. The study revealed that various cell types respond differently to osmotic stress, either through specific TRNs regulated by transcription factors or through cis-regulatory elements linked to stress-related genes (gl-cCREs), enabling them to adapt effectively. Additionally, the researchers identified potential target genes and stress-related gl-cCREs, highlighting significant cellular heterogeneity in response to osmotic stress.

In a study of gene function, Turco et al. employed Drop-seq technology to produce comprehensive individual cell data on entire roots of *Arabidopsis* [5]. Their findings revealed that the activation of *VND7* triggers a rapid transition from root cell to xylem cell characteristics. Furthermore, they successfully identified four potential target genes regulated by *VND7* that have the ability to induce this transformation. Through the utilization of an extensive single-cell gene expression atlas for roots of *Arabidopsis*, Wendrich et al. observed an increased presence of *Target of monopteros 5/Lonesome highway (TMO5/LHW)* responses, specifically within cells in root hair [58]. The researchers provided evidence supporting the role played by the *TMO5/LHW* heterodimer in stimulating the production of mobile cytokinins within vascular cells. Additionally, they found that this process leads to an augmentation in root hair density when subjected to a low-phosphate treatment through alterations in both epidermal cell length and fate determination. Han et al. utilized the SingleCellGGM algorithm to analyze publicly accessible scRNA-seq data of *Arabidopsis* roots [61]. Through the investigation, they successfully detected 149 unique gene expression programs (GEPs). Furthermore, by applying advanced spatiotemporal expression mapping methods, the researchers accurately outlined diverse gene expression programs that regulate the development and differentiation of distinct types of root cell during various developmental stages.

#### 3.1.2. Research on Leaf Tissue

Liu et al. conducted a comprehensive study on the growth of stomatal lineage cells using an scRNA-seq analysis [63]. They focused on five-day-old cotyledons of *Arabidopsis* seedlings to identify distinct cellular categories and their corresponding marker genes in stomatal lineage cells. Additionally, they investigated the transcriptional networks responsible for regulating the transition from meristemoid mother cells to guard mother cells. Kim et al. focused on enhancing vascular cells derived from *Arabidopsis* leaves to create an extensive atlas detailing the transcriptomes of individual cells within leaf vasculature [66]. Their analysis revealed a minimum of 19 distinct cellular clusters, shedding light on various aspects related to leaf vasculature as well as elucidating the roles and interconnections among different types of leaf cells. Meanwhile, Lopez-Anido et al. employed an integrated approach combining scRNA-seq with molecular genetics to investigate how cell differentiation occurs in *Arabidopsis* leaves [65]. Notably, they discovered novel models that explain this process within the tissue structure while also highlighting the significance of SPEECHLESS, a transcriptional regulator, in reinforcing cell fate determination. Liu et al. developed a detailed transcriptome framework for de novo root regeneration (DNRR) from *Arabidopsis* leaf explants, using a time-lapse and scRNA-seq data [62]. They identified the key factors involved in this process. In another study, Zhu et al. investigated the response of *Arabidopsis* leaves to a *Pseudomonas syringae* infection or a control treatment [64]. By analyzing over 11,000 cells with scRNA-seq, they observed distinct cell populations representing immune and susceptible states. The researchers also found that the size of bacterial colonies and the duration of infection influenced the distribution of these cell populations. Furthermore, their work provided valuable insights into studying plant host–pathogen interactions at a high-resolution level.

#### 3.1.3. Research on Other Tissues

Song et al. employed scRNA-seq to investigate the correlation between ploidy, cell size, and transcriptome abundance in *Arabidopsis* tetraploid lines and isogenic diploids [67]. Their findings revealed that tetraploid plants exhibited a twofold increase in transcriptome abundance per individual cell compared to diploid plants. However, the extent of this change and its association with cell size varied depending on the specific type of cell analyzed. Zhang et al. employed scRNA-seq to characterize the cellular classification within the *Arabidopsis* vegetative shoot apex, based on transcriptomic data [54]. By identifying various cellular groups and changes in states, they elucidated how cell division and differentiation within the shoot apical meristem enable the repetitive development of new aboveground structures with a high-resolution examination at individual cell level. In another investigation by He et al., a comprehensive organism-wide cell atlas was constructed by integrating 28 previously published scRNA-seq datasets obtained from young *Arabidopsis* seedlings [70]. This work further delved into analyzing specific lncRNA signatures related to different cell types, while also mapping out the transcriptional diversity among lncRNAs, along with their interconnected gene regulatory networks, using single-cell resolution techniques.

Sunaga-Franze et al. introduced an snRNA-seq methodology utilizing the nanowell-based ICELL8 system to examine the spatiotemporal dynamics of *Arabidopsis* transcriptomes during flower development [69]. They acknowledged that by focusing on nuclei, they could effectively exclude organelles, vacuoles, and cytoplasmic secondary metabolites that may potentially interfere with RNA interactions and impede the next-generation sequencing (NGS) library preparation. In the same year, Farmer et al. reported a study on *Arabidopsis* roots in which they utilized snRNA-seq [59]. They compared the transcriptomes of single nuclei with previously published protoplast transcriptomes, confirming that plant cell type-specific transcriptomes can be established by using nuclei as biological entities. Additionally, the application of snRNA-seq uncovered the presence of previously unidentified cell subtypes that were not detected using scRNA-seq.

### 3.2. Research and Application in Crops

According to their agronomy and use, crops are mainly divided into food crops, economic crops, forage and green manure crops, and medicinal crops. Currently, the scRNA-seq technology is applied to food crops, economic crops, and forage and green manure crops.

#### 3.2.1. Research on Food Crops

Food crops are mainly used as food and include three major groups: cereals, legumes, and potato and taro crops. scRNA-seq studies of cereal crops, such as maize (*Zea mays*), rice, wheat (*Triticum aestivum*), the legume crop soybean, and the potato and taro crop cassava (*Manihot esculenta*), have been reported in the past few years.

In the field of maize research, scRNA-seq is primarily used to reveal the developmental programs of different tissues, construct transcriptional regulatory networks, and identify key genes or regulators. In 2019, Nelms and Walbot were pioneers in employing scRNA-seq to analyze the progression of male meiosis during maize development [71]. They introduced a quantitative methodology known as “pseudotime velocity” to deduce developmental shifts by identifying rapid alterations in gene expression. This framework was then applied to identify intermediate cellular stages during germinal development. Satterlee et al. employed scRNA-seq to obtain an impartial understanding of the transcriptional profile in the stem cell niche of maize shoots and its subsequent differentiating cells [72]. The acceleration of cell differentiation and the promotion of growth in the sheathing leaf base of maize are facilitated by the increased expression levels of *KNOTTED1 (KN1)*. Xu et al. constructed and validated a detailed transcriptome atlas of maize ear inflorescence development [73]. They also established transcriptional regulatory networks at the cellular level and identified potential loci linked to ear yield traits. In another study, Zhang et al. sequenced the transcriptomes of individual meiocytes during the tetrad stage and of early mononucleate microspores from both sterile and restorer lines [74]. They found 3379 genes with differential expression, including 277 genes potentially associated with mitochondria, 226 genes encoding transcription factors, and 467 genes that may be targeted by RF4. The researchers demonstrated that the surrounding cells, as well as the restorer gene, influenced meiocyte and microspore development by restoring redox homeostasis in microspores while facilitating normal cellular reconstruction during transition periods. Marand et al. provided a comprehensive analysis of the cis-regulatory landscape in maize, a genetically modified crop species, at the single-cell level [75]. By taking an evolutionary perspective, they discovered that decayed long terminal repeat (LTR) retrotransposons play a role in shaping cell type-specific genetic networks. Additionally, they identified specific cis-regulatory elements (CREs) associated with alleles targeted by modern breeding practices and assessed how variations in cis-regulatory regions have influenced cellular differentiation between two distantly divergent angiosperms. Li et al. conducted a comparison of scRNA-seq profiles for various cell types under varying nitrate conditions, leading to the identification of four genes associated with the uptake and utilization of nitrates [76]. By comparing root cells from maize and rice, researchers discovered both commonalities and variations in terms of cell types and characteristics. Sun et al. employed fluorescence-activated sorting to isolate propidium iodide-stained nuclei from maize epidermal peels, subsequently establishing a high-capacity method for conducting snRNA-seq analyses [77]. Utilizing this methodology, researchers examined a sample taken from the base of a maize leaf to uncover novel potential factors contributing to the development of grass stomata. Within this group of potential contributors, researchers pinpointed specific genes related to cell walls that could potentially exert a significant influence on both the initial formation as well as the subsequent maturation processes associated with dumbbell-shaped stomata. In the study published in 2024, Yuan et al. utilized scRNA-seq along with optimized DNA affinity purification sequencing using a PCR-amplified genomic DNA library known as ampDAP-seq [78]. This approach allowed them to create a comprehensive transcriptome atlas and wide-ranging TF-DNA-binding patterns in developing maize endosperms during the process of cell differentiation. They mapped regulons to endosperm cell clusters and successfully identified crucial regulators that are unique to each cell cluster. To validate their findings, they conducted experiments that confirmed the significance of three predicted key regulators named EREB108, MYBR19, and MYBR29. Additionally, researchers have made these valuable datasets accessible through an online interface available at https://www.maize-endosperm.cn/ (accessed on 25 July 2024). Wang et al. performed a comprehensive transcriptomic mapping of the ligular region using both bulk and snRNA-seq techniques [79]. They discovered a large number of genes that are highly expressed in hypodermal cells, potentially influencing their specialization in sclerenchyma cells. Additionally, they investigated two untypical basic helix–loop–helix (bHLH) TFs, namely bHLH30 and its homolog bHLH155. These factors were found to play a role in regulating plant architecture by activating the transcription of genes responsible for the elongation and lignification mechanisms in sclerenchyma cells. Another study on leaves identified abaxial bundle sheath cells as being specialized in nutrient transport. Transcripts of SWEET13a, b, and c, as well as other sugar and amino acid transporters, were enriched in the abaxial bundle sheath cells [80].

In the realm of rice research, scRNA-seq technology offers valuable insights into the transcriptomic makeup of individual cells and sheds light on the developmental trajectory of important organs such as roots, leaves, and inflorescences. In a recent study by Zhang et al., they employed scRNA-seq and chromatin accessibility analyses to investigate rice radicles, successfully confirming the presence of 21 distinct cell types in the root tip [12]. Through the temporal profiling of these specific cell populations, they were able to reconstruct the sequential development patterns of epidermal cells and ground tissues while uncovering potential regulatory networks involved in determining cell fate within these lineages. Additionally, they identified characteristic processes, transcriptome profiles, and marker genes associated with each cell type, revealing both conserved and divergent pathways governing root development between dicots and monocot. Liu et al. covered transcriptomes of more than 20,000 individual cells obtained from the root tips of two important rice cultivars, namely Nip and 93–11 [9]. They successfully identified the primary cell types and established unique marker genes specific to each cultivar. Notably, they uncovered a wide range of cell types associated with distinct regulatory programs such as phytohormone biosynthesis, signaling, and response. These regulatory programs exhibited remarkable conservation between the two rice cultivars. However, notable differences were observed in the cell-type transcript profiles when comparing *Arabidopsis* and rice. Wang et al. conducted an scRNA-seq analysis on rice seedlings’ shoots and roots, which were either grown in Kimura B nutrient treatment or subjected to different abiotic stresses [10]. In total, 237,431 cells were obtained from this study. Moreover, they characterized fifteen distinct cell categories in leaves and nine cell categories in roots. Furthermore, they revealed three fundamental principles governing how plant cells respond transcriptionally to abiotic stresses: these responses are often specific to particular cell types, exhibit some degree of similarity across various abiotic stresses, and occasionally show similarities between corresponding cells in leaves and roots. Xie et al. established a straightforward approach to predict the targets of transcription factors (TFs) in rice leaf cells using the 10× Genomics scRNA-seq technique [11]. By analyzing expression profiles, they identified 35 potential targets that exhibited strong correlations with the TF OsNAC78 expression. Among these targets, Os01g0934800 and Os01g0949900 showed remarkably similar expression patterns to OsNAC78 and were determined as its targets. Zong et al. utilized scRNA-seq to analyze 37,571 cells from rice inflorescences and created a comprehensive gene expression database that covered the progression from inflorescence to floret during the initial phases of reproductive growth [81]. They reconstructed the developmental paths of both florets and meristems, identifying distinct cell types as well as regulatory factors within the diverse population of young inflorescences. Moreover, they presented evidence supporting the role of a WOX TF (DWARF TILLER1) in regulating flower meristem activity. Furthermore, they investigated the expression patterns and functional characteristics of OsAUX1, an auxin transporter, shedding light on its involvement in rice inflorescence branching. Li et al. utilized snRNA-seq to construct a thorough spatiotemporal survey of rice pistils before fertilization [82]. They also revealed the cellular composition of ovule- and carpel-derived cell trajectories at the individual cell level.

In addition, Zhang et al. performed snRNA-Seq and ATAC sequencing on the roots of bread wheat to investigate asymmetrical gene transcription and to reconstruct cellular differentiation trajectories as well as cell type-specific gene regulatory networks [83]. They also explored the distinct origins of the epidermis/cortex and the endodermis in bread wheat compared to *Arabidopsis*. Additionally, *TaSPL14* plays a role in vascular development by regulating *BAM1* expression. Song et al. performed scRNA-Seq at two different developmental stages in cassava tuberous roots, successfully categorizing a total of 14,566 cells into 15 unique cellular categories [84]. They also identified a unique cellular subtype, called Casparian strip, by examining genes that are upregulated and conducting a pseudotime analysis, shedding light on its differentiation process from the endodermis.

The legumes establish a symbiotic relationship with rhizobium, resulting in the formation of nitrogen-fixing nodules. In soybean, scientists tend to use snRNA-seq in conjunction with other technologies to characterize the transcriptomic profile of different cell types in soybean roots and nodules. Sun et al. successfully constructed a comprehensive transcriptomic atlas of soybean roots and nodules at the individual nucleus level and identified 17 cell types, among which, six are exclusive to nodules [16]. They also investigated the developmental process of soybean nodules, which exhibited distinct characteristics compared to the indeterminate nodules found in *M. truncatula*. Additionally, they highlighted the significance of *GmCRE1*, a cytokinin receptor, in nodule formation and biological nitrogen fixation. Furthermore, several potential regulators involved in soybean nodulation were identified. Among them, there were two previously uncharacterized genes in soybean: *GmbHLH93* and *GmSCL1*. Another study on soybean nodules employed a combination of single-nucleus and spatial transcriptomics to construct a comprehensive cell atlas of nodules and roots [15]. The findings indicated that uninfected cells exhibited specialization in distinct functional subcategories during nodule development, while also revealing a transitional subtype of infected cells enriched with genes related to nodulation. Utilizing snRNA-seq and Molecular Cartography^TM^ techniques, the transcriptomic profile of distinct cell types in the root and mature nodule of soybean was examined. This analysis unveiled a range of subgroups within mature soybean nodules that were infected by *B. diazoefficiens*. Additionally, this study validated the involvement of known genes associated with nodulation and identified novel genes, such as *GmFWL3,* that regulate the process of nodulation [14]. Recently, Zhang et al. utilized ten soybean tissues as experimental samples to generate high-resolution single-cell gene expression atlases [17]. By employing snRNA-seq, ATAC-seq, and spatial transcriptomics techniques, they identified a total of 103 cell types and 303,199 accessible chromatin regions (ACRs). This comprehensive study unraveled intricate gene networks and developmental transitions within the soybean system. Notably, their findings revealed that approximately 40% of the ACRs displayed patterns specific to certain cell types and had a notable enrichment of TF binding motifs linked to those particular cell types. Moreover, to learn how nutrient stress impacts plant growth and improve crop production under nutrient limitation, Chen et al. applied scRNA-seq to shoot apices of *Pisum sativum* (pea) grown under a boron (B) deficiency. They found that the response of shoot apices to a deficiency in B displays significant heterogeneity, with different cell types showing diverse responses that are associated with their specific functions [85].

#### 3.2.2. Research on Economic Crops

Several current studies have put scRNA-seq/snRNA-seq into use for economic crop research, such as the fiber crop cotton (*Gossypium hirsutum*), the vegetable crops Chinese cabbage (*Brassica rapa*) and tomato (*Solanum lycopersicum*), the oil crop peanut, and the stimulant crop tobacco (*Nicotiana attenuata*).

Cotton fibers are derived from the ovule epidermis, consisting of a single cell, making it an ideal system for investigating the mechanisms involved in determining cellular destiny. Qin et al. utilized scRNA-seq to detect a total of 14,535 cells across four developmental stages within the outer integument of the Xu142_LF line [86]. Through their analysis, they successfully distinguished three primary cell types, namely fiber cells, non-fiber epidermis cells, and outer pigment layer cells. Additionally, their investigation revealed interesting insights into the temporal–spatial expression patterns of key TFs. Notably, they observed that *MYB25-like* and *HOX3* are crucial regulators in governing both fiber differentiation processes and tip-oriented diffuse growth. Thereafter, using integrated scRNA-seq and scATAC-seq, Wang et al. further identified two cardinal cis-regulatory elements (TCP motif and TCP-like motif) which are bound by the transfactor *GhTCP14s* to modulate the circadian rhythmic metabolism of mitochondria and protein translation through regulating approximately one-third of the genes that are highly expressed in fiber cells [87]. Zhu et al. generated a detailed transcriptomic map of individual cells in the hypocotyl tissue from two cotton genotypes: Jin668, which has high regenerative capacity, and TM-1, which is less responsive to regeneration [88]. They further identified 41 genes associated with hormone response, such as *LAX2*, *LAX1*, *LOX3*, and also identified novel genes involved in regeneration, such as *CSEF*, *PIS1*, *AFB2*, *ATHB2*, *PLC2*, and *PLT3*. Cottonseeds, which are regarded as the byproducts of fiber production, are rich in unsaturated fatty acids, proteins, and vitamins. Nevertheless, the exploitation of cottonseed for food is restricted because of the existence of pigment glands containing gossypol and its derivatives, which possess toxicity towards humans. To discover novel regulators implicated in the formation of pigment glands, Long et al. conducted single-cell RNA sequencing on cotyledons from two different types of cotton plants: “CCRI12”, with pigment glands, and “CCRI12gl”, without pigment glands [89]. After filtering, a total of 9186 individual cells were obtained and classified into 12 clusters based on the genes that showed high variability. The authors suggest that *GhJUB1* may play a role in regulating the formation of pigment glands downstream of *GoPGF* (*Gossypium PIGMENT GLAND FORMATION*). Another study was conducted on pigment glands, focusing on 12,222 protoplasts that were extracted from the cotyledons of sprouting seeds [90]. These cells were clustered into 14 distinct clusters in an unsupervised manner and categorized into eight cell populations by utilizing known cell marker genes. Subsequently, a model for the development of pigment glands was established, with light and gibberellin being confirmed as promoters in this model. Furthermore, they identified three new genes, *GbiERF114*, *GbiZAT11*, and *GbiNTL9*, that can affect the development of pigment. A comprehensive analysis of the transcriptome profile in cotton leaves was conducted by Lin et al., who also established a hierarchical transcriptional network that controls the production of cell-specific terpenoids using scRNA-seq [91]. Two previously unidentified TFs, called GoHSFA4a and GoNAC42, were identified as the downstream effectors of the *GoPGF* gene. These TFs play a direct role in regulating the expression of genes associated with terpenoid biosynthesis in secretory glandular cells, exhibiting responsiveness to both developmental signals and environmental stimuli. Li et al. constructed a comprehensive single-cell transcriptome atlas and conducted a chromatin accessibility survey of cotton anthers at the tetrad stage, both under normal and high temperature conditions [92]. The distinct expression patterns and epigenetic profiles observed in meiotic, tapetal, and middle layer cells within anthers at normal temperature strongly support the notion of functional specialization among these cell types.

The utilization of scRNA-seq can aid in comprehending the prevalence of gene expression and provide fresh insights into maintaining gene dosage equilibrium within the subgenome at a cellular level in Chinese cabbage. Sun et al. employed scRNA-seq to analyze 30,000 individual cells and obtained a detailed transcriptional profile of Chinese cabbage leaves [93]. Their findings revealed that genes belonging to the least-fractionated subgenome exhibited higher levels of expression compared to those from the medium- and most-fractionated subgenomes across various cell types. Additionally, they constructed a single-cell transcriptional map depicting leaf responses under high temperature conditions, which demonstrated that the effects of heat stress on gene expression vary depending on cell type and also influences subgenome predominance. Guo et al. used protoplasts derived from the rosette stage young Chinese cabbage leaves to conduct scRNA-seq, resulting in the creation of a cellular-level transcriptome atlas for the rosette leaves of Chinese cabbage [94]. A notable discovery was made regarding the differentiation between adaxial palisade mesophyll cells (PMCs) and abaxial spongy mesophyll cells (SMCs) within the broader category of mesophyll cells (MCs). Furthermore, they unveiled distinct functional variations between PMCs and SMCs and highlighted the potential involvement of genes encoding ribosomal proteins in PMCs development.

In tomatoes (*Solanum lycopersicum*), Tian et al. demonstrated a robust method with minimal artifacts for snRNA-Seq, which can be widely utilized for other species and tissues [95]. They generated a high-resolution expression atlas of shoot apex cells, identified crucial developmental regulators, and determined the cell hierarchy. Song et al. conducted snRNA-seq and spatial transcriptomic analysis on tomato calli grown in a regeneration medium, uncovering the existence of diverse cell populations within the calli. Chlorenchyma cells, known for their involvement in photosynthesis-related processes, contribute significantly to the facilitation of shoot primordia formation and subsequent shoot regeneration [96].

In a study on peanuts, Liu et al. initially isolated 6815 individual cells and categorized them into eight distinct cell clusters based on the identification of marker genes using scRNA-seq. Then, a single-nucleus atlas of peanut leaves was developed by simultaneously profiling the transcriptome and chromatin accessibility in the same individual cell using a fluorescence-activated, sorted single nuclei [23,27]. Du et al. isolated *fad2* (*fatty acid desaturase*) mutant leaf protoplast cells to carry out scRNA-seq [24]. A total of 24,988 cells were identified, with 10,249 genes being expressed across five primary cell categories. Through a comprehensive examination of 3495 genes that exhibited differential expression in various cellular types, it was observed that *fad2* suppressed the activity of the *LOG* gene responsible for cytokinin synthesis in vascular cells, consequently impeding leaf development. By analyzing single cells from peanut seedling leaves under both dark and light conditions (13,409 and 11,296 cells, respectively), Deng et al. characterized ten cell clusters using previously identified scRNA-seq marker genes [25]. In total, 6104 genes and 50 TFs exhibited significant expression patterns in specific cell clusters. Among these, peanut *AHL17*, which encodes a nuclear protein, promoted leaf epidermal cell enlargement when artificially overexpressed in *Arabidopsis* through the modulation of a regulatory phytohormone pathway. To elucidate the molecular mechanisms underlying peanut pod development, Cui et al. characterized the cellular anatomical features of aerial peg, subterranean peg, and expanded pod [26]. They employed a comprehensive approach, integrating snRNA-seq and snATAC-seq data to investigate gene expression dynamics and chromatin accessibility during peanut peg insertion into soil and subsequent pod expansion at a single-cell resolution. Through the integration of in situ RNA hybridization validation and a pseudotime analysis, they unveiled crucial gene regulatory networks involved in the gravity response of pegs, as well as the transition from peg insertion into soil to pod enlargement.

In addition, Kang et al. employed the scRNA-Seq technique to analyze protoplasts derived from the corolla limbs and throat cups of tobacco (*Nicotiana attenuata*) [97]. Their study aimed to elucidate the complete biosynthetic pathway of a floral volatile compound and to identify the distinct cell clusters responsible for benzylacetone synthesis, which is an essential component of flower scents, based on variations in gene expression at the cellular level.

#### 3.2.3. Research on Forage and Green Manure Crops

*Lotus japonicus* is a significant perennial legume used as a model organism in investigating symbiotic nitrogen fixation. To unravel the function of various cell types of roots and understand root development and nodulation at single-cell resolution, Sun et al. [98] performed scRNA-seq and profiled 22,688 single root cells. They identified seven major cell types and revealed the regulatory programs associated with specific cell types, including phytohormone and nodulation, and even uncovered some conserved and diverged features for the cell types. Thereafter, Wang et al. [100] identified specific genes that contribute to the distinct activities of infected cells and uninfected cells in N_2_-fixing nodules, including *LjAMT1.1*, *LjERF1*, *Lj4g3v2215210.1*, *Lj3g3v0323220.1*, and so on. Frank et al. [99] further identified *SYMRKL1*, which codes for a protein that possesses an ectodomain, exhibiting a high similarity to that of SYMRK, and plays a crucial role in the formation of infection threads.

*M. truncatula*, a legume species widely investigated over many years, also serves as an invaluable model organism for comprehending the mutualistic association between legumes and rhizobia, a group of soil bacteria. Cervantes-Pérez et al. focused on analyzing the response of *Medicago* roots to rhizobial infection [20]. They utilized nuclei from both untreated and rhizobia-inoculated roots for snRNA-seq profiling, aiming to gain a better understanding of the initial reactions triggered by rhizobial infection in *Medicago* roots. Ye et al. exploited an improved method for preparing protoplasts from nodules and conducted a scRNA-seq experiment on a type of nodule found in *M. truncatula* that is not well understood [18]. They identified 13 distinct clusters of cells within the nodules based on their unique gene expression patterns and constructed a comprehensive map detailing the spatial distribution and functional characteristics of these cells. Through a pseudotime analysis and a biofunction examination, it was demonstrated that the two groups of apical meristematic cells follow distinct developmental trajectories, leading to either a symbiotic or non-symbiotic fate, while uninfected cells located in the nitrogen fixation zone play a crucial role in nitrogen assimilation by actively participating in the asparagine synthesis pathway. To investigate the initial cellular responses to nodulation, Liu et al. conducted a time–course analysis using snRNA-seq on *M. truncatula* roots after treatment with nod-factor [19]. They observed significant global gene expression changes in both the epidermis and the cortex within 30 min, which were mostly reversed by 6 h. Additionally, the activation of genes related to the defense response in plants occurred at 30 min, followed by their suppression after 6 h in non-meristem cells. Furthermore, they discovered that *MtFER* and *LYK3* exhibited comparable reactions to symbiotic signals and that *LYK3* was able to phosphorylate *MtFER*, thereby participating in rhizobial symbiosis. Recently, Pereira et al. [21] and Serrano et al. [22] provided insight into a symbiotic relationship of major agricultural and environmental importance and even demonstrated a paradigm of multiple omics for the analysis of complex organismal interactions.

### 3.3. Research and Application in Woods

The application of scRNA-seq technology has significantly transformed our comprehension of the expression patterns of genes within individual cells. Utilizing the scRNA-seq system to investigate wood-forming tissues at the cellular level enables the discovery of diverse cell transcriptomes, which can be categorized into distinct clusters.

scRNA-seq of protoplasts derived from the differentiating xylem of *Populus alba*×*Populus glandulosa* demonstrated the presence of 12 unique cell clusters within the xylem tissue, encompassing vessel cells, fiber cells, ray parenchyma cells, and xylem precursor cells. Differentiation processes between vessels and fiber cells were found to be distinct based on diffusion pseudotime analyses, while a similar differentiation process was observed between fiber cells and ray parenchyma cells [101]. By using multiple moduli for the identification of cell clusters, it becomes possible to allocate the same tissue to various clusters. In another study, a refined method for isolating protoplasts and RNA sequencing were employed to construct a detailed single-cell transcriptional map of highly lignified poplar stems [102]. The researchers discovered 20 distinct cell clusters characterized by unique marker genes, indicating significant heterogeneity in these cells based on their transcriptome profiles. Furthermore, they observed that different cell clusters displayed specific patterns of phytohormone responses. They also reconstructed cell differentiation trajectories with reference to phloem and xylem development. Through both scRNA-seq and laser capture microdissection transcriptomic profiling techniques, Tung et al. verified the developmental lineages of ray and fusiform cells among four distinct woody angiosperms [103]. Consequently, it has been observed that the radial and axial systems within the stem-differentiating xylem undergo development via two cell lineages, ultimately leading to terminally differentiated cells. Furthermore, their findings suggest that tracheids exhibit a transcriptomic profile more closely resembling vessel elements, rather than libriform fibers, due to the absence of expression of *SND2* genes in vessel-less seed plants. Li et al. employed scRNA-seq and spatial transcriptome sequencing techniques to uncover the transcriptional profiles of primary and secondary growth samples within *Populus* stems [104]. They discovered distinct regulatory networks governing cell differentiation from cambium to xylem and phloem precursors, which potentially be influenced by the accumulation and distribution of auxin. An examination of cell differentiation trajectories also indicated a sequential trend in transcriptional regulation during vessel and fiber development. Furthermore, using single-cell sequencing as a tool, Zhang et al. identified PagMYB31 as a coordinator, regulating wood formation processes in *Populus alba × Populus glandulosa,* and built a PagMYB31-mediated transcriptional regulatory network [105].

## 4. Some Databases for Marker Gene Searching in Plants

The identification of each cluster’s cell identity can be achieved by examining the expression patterns of specific genes, which necessitates prior knowledge regarding gene expression in the relevant tissues or species [2]. However, it remains challenging to accurately annotate and complete this step due to the lack of well-established marker genes for various cell categories and states [106]. To help overcome this difficulty, in the following paragraphs, we introduce databases from which a great deal of information about marker genes in common plant tissues can be obtained.

PlantscRNAdb, PsctH (Plant Single Cell Hub), PCMDB (The Plant Cell Marker DataBase), scPlantDB, and PlantPhoneDB are five comprehensive databases dedicated to the single-cell RNA analysis of plants that contain marker genes of different cell categories in the diverse tissues of various plant species (Table 2). They additionally offer entry to the primary dissertations and linked expression matrices, along with graphical depictions of gene expression using t-SNE or UMAP [1].

PlantscRNAdb is a comprehensive repository for plant single-cell RNA analyses that collected 15 species of single-cell or single-nucleus RNA data and four spatial transcriptomics datasets from recently published articles for Release 3.0. The database can also be used to explore gene expression of the single-cell type and at the genome-wide scale. A total of 112,657 marker genes identified from 15 species in a single-cell RNA analysis and 11,809 marker genes identified from four species of spatial transcriptomics data were identified in this database. Furthermore, the database categorizes the marker genes into two grades (marker #1 and marker #2) based on their expression pattern across different cell types. [107]. PCMDB uses normal selection criteria to offer a comprehensive summary of cellular indicators from experimental studies, bulk RNA-seq, and scRNA-seq studies. It possesses six model plants with three different type cell markers, including 3119 experimentally validated marker genes collected by manual curating from 1676 papers, 40,625 DEGs based on bulk RNA-seq data, as well as 46,915 DEGs across particular cells, confirmed by scRNA-seq. Information including tissue type, sub-tissue type, and cell type was recorded. In addition, it supports different analysis tools, including SingleR, SCSA, and BLAST, which have the potential to facilitate cell type prediction and cell marker prediction based on marker gene information [108]. PsctH is an open-access database of high-throughput sequencing data of plant single cells and has collected 16 plant scRNA-seq raw data from five species. Altogether, the set of 104 marker genes, identified by RNA in situ hybridization and GFP reporter methods, is available to download. In addition to identifying marker genes, a practical protocol was provided for guiding the preparation of dissociated protoplasts. Additionally, a comprehensive pipeline using R scripts was developed for facilitating plant scRNA-seq analyses [109]. PlantPhoneDB is an extensive pan-plant database that includes numerous highly reliable ligand–receptor pairs, manually filtered from seven different sources. Furthermore, the database comprehensively compiled information on 29 single-cell datasets, encompassing 15 distinct tissue types across five species, encompassing crucial details such as experimental protocols, cell quantification, and sequencing platforms. They also developed an R package that offers not only four optional scoring methods for calculating interaction scores between cell types in ligand–receptor pairs, but also visualization functions to demonstrate the analysis findings. The PlantPhoneDB web interface allows users to search, browse, or download processed datasets and results [110]. scPlantDB is a comprehensive database designed to investigate plant cell atlases, allowing researchers to explore cell types and markers. It integrates transcriptomic data from 67 different datasets, covering 17 plant species, encompassing around 2.5 million individual cells. The platform provides the interactive visualization of gene expression at the cellular level, allowing for the analysis of single and multiple datasets. Additionally, scPlantDB facilitates the systematic evaluation and functional annotation of markers across diverse cell types and species. It also provides resources for the identification and comparison of cell types based on these marker genes [111]. The number of species and marker genes contained in the aforementioned databases differs, thereby enabling resource complementarity. Multiple databases provide marker genes and other information, serve as complementary approaches, and can be utilized for cell type identification, systematic comparisons, and functional annotations. However, the absence of a centralized database for storing and sharing single-cell sequencing data currently presents challenges in data processing due to redundancy issues.

## 5. Perspectives

Plant cells serve as the essential components that underpin plant growth and development, and a comprehensive and in-depth description of plant cell characteristics can help solve problems in plant science. For this reason, scientists first proposed the Plant Cell Atlas (PCA) project in 2019, which seeks to build a comprehensive information database of all cell types, not only providing data on the level of gene expression in every cell type, but also revealing the specific functions of each cell type in signal transduction, cell differentiation, and plant metabolism. The advent of single-cell omics techniques and their recent implementation in various plant organs has facilitated the analysis of numerous cellular properties within a single study [106,112] and can even help scientists obtain cell type information, analyze the information, and collect valuable insights for PCA projects.

scRNA-Seq has been employed in the investigation of plant-specific cell types and many other biological processes. For example, several single-cell transcriptome maps have been constructed for *Arabidopsis* lateral roots [55,57], vegetative shoot apices [54], blade vascular systems [66], and other tissues to explore biological problems [68,113]. Pseudotime analysis holds the capability to predict the developmental trajectories of particular cells in plant tissues, such as revealing leaf developmental changes in *Arabidopsis* [65], predicting the formation of lateral root quiescent central cells in *Arabidopsis* [55,56], and establishing the developmental trajectory of inflorescences in rice [81]. Furthermore, scRNA-seq has the potential to be utilized in examining the reactions of plant cells to biotic and abiotic pressures [93,114], exploring the similarities and distinctions among cell types across various species [12,76,103,115], and screening high-quality genes for crop breeding [73]. Nevertheless, there remain numerous obstacles in the field of plant single-cell transcriptomics, resulting in a predominant emphasis on generating single-cell transcriptome maps and cellular development trajectories solely for *Arabidopsis*, rice, and other model crops. First, protoplast-based protocols are widely used for plant scRNA-seq. Plant protoplasts larger than 100 μm in diameter may lead to cell breakage and equipment plugging. In addition, the efficiency of protoplast preparation in plant cells is influenced by various factors, such as cell wall composition and tissue location, which differ from animal cells due to the presence of cell walls. Enzymatic digestion may introduce a noticeable bias in the expression of relevant genes. To address this issue, some studies have opted for snRNA-seq instead of scRNA-seq as it allows access to a broader range of cell types and offers advantages in analyzing diverse tissues and species, even eliminating potential capture bias [95]. scRNA-seq requires cell lysis, which hinders forward molecular and functional analyses of the same cells. Therefore, only the static state of the cell can be measured, and the dynamic state of the cell cannot be continuously observed using scRNA-seq. Fortunately, to tackle this problem, Live-seq utilizes fluidic force microscopy for the extraction of RNA while simultaneously preserving cell viability to enable single-cell transcriptome profiling [42]. Nevertheless, scRNA-seq provides the depth of the transcriptome, while snRNA-seq provides the breadth of the transcriptome; when these two techniques are combined and analyzed together, they offer complementary insights into plant cell identity [116]. Second, alignment failure may occur due to the utilization of reference genomes of low quality, resulting in the omission of expression signals from specific genes. Hence, it is imperative to have access to high-quality reference genomes for single-cell transcriptomics. The trend of scRNA-seq in plants extends from model plants to other plants to enable multi-tissue and multi-sample research. Third, because the study of plant single cells is currently at an initial stage, the accuracy of cell type annotation in many species is incomplete, which hampers the use of this technology in various species. In recent years, with deepening research on scRNA-seq in various species, the characteristic expression genes of cell subsets in model plants, for example *Arabidopsis* and rice, have been matched, and online databases have been constructed (Table 2), which will help in cell type annotation. In addition, bioinformatics methods such as cross-species searches for homologous genes can be used to locate marker genes. Furthermore, experimental methods, for example in situ hybridization and immunofluorescence, are usually employed to verify the accuracy of marker genes, although they may be more time-consuming [1].

The field of multiomics is continuously evolving, as scRNA-seq is being integrated with scATAC-seq, spatial transcriptomics, and mass spectrometry imaging (MSI). By employing single-cell ATAC-seq, researchers can investigate the DNA regions that encompass the regulatory codes responsible for the observed transcriptomic patterns in scRNA-seq data [112]. Combining scRNA-seq with scATAC-seq allows for the simultaneous capture of unique cell transcriptomes and epigenomes. This approach provides a wealth of data with which to depict gene expression patterns, identify cell categories, and investigate the collaborative role of different transcription factors in regulating gene expression networks [117]. A drawback of scRNA-seq is the absence of spatial data regarding precise cellular localization within a tissue during cell isolation [118]. However, through the integration of scRNA-seq data with spatial transcriptomic techniques, it becomes possible to reconstruct the spatial and temporal characteristics of cell types and states within a tissue. Although spatial transcriptomics techniques offer a solution to the limited availability of spatial data in scRNA-seq, their current inability to achieve single-cell resolution and difficulties in breaking down plant cell walls and facilitating the diffusion of intracellular transcripts onto array surfaces have hindered their widespread adoption in plant research. However, there have been some notable exceptions where these techniques have been successfully applied, such as studying *Arabidopsis* inflorescence meristem, *Picea abies* female cones, and *Populus tremula* leaf buds [119]. MSI is a cutting-edge technique in molecular imaging that allows for the detection of the spatial arrangement of numerous metabolites within specific tissues. This advanced technology allows for the rapid acquisition of extensive data regarding the composition, structure, and spatial arrangement of molecules within living tissues. It encompasses both identified and unidentified endogenous metabolites, facilitating the generation of detailed maps depicting tissue-specific molecular patterns [120,121]. Owing to the improvement of spatial resolution based on MSI technology, it can be used to explore the biological functions of primary and secondary metabolites within tissues, which will represent the regulatory mechanisms at the cellular or subcellular levels during plant growth. Compared with animal, medical, and other fields, there are still many challenges in plant scRNA-seq technology; however, there is no doubt that the application of scRNA-seq and multiomics technologies will bring a new vision for the fine study of plant cells, especially as an effective way to study plant tissue development.

## Figures and Tables

**Table 2:** Databases for plant marker gene searching.

Database	Website	Species	Number of Maker Genes	Last Modified Date	Reference
PlantscRNAdb	http://ibi.zju.edu.cn/plantscrnadb/index.php (accessed on 25 July 2024)	*Arabidopsis thaliana*, *Oryza sativa*, *Solanum lycopersicum*, *Zea mays*, *Fragaria vesca*, *Populus*, *Nicotiana attenuata*, *Lemna minuta*, *Brassica rapa*, *Manihot esculenta*, *Medicago truncatula*, *Nepeta tenuifolia*, *Gossypium hirsutum*, *Glycine max*, *Phalaenopsis aphrodite*	114,770	15 August 2023	[107]
PCMDB (Plant Cell Marker Data Base)	https://www.tobaccodb.org/pcmdb/homePage (accessed on 25 July 2024)	*Arabidopsis thaliana*, *Oryza sativa*, *Zea mays*, *Glycine max*, *Solanum lycopersicum*, *Nicotiana tabacum*	81,117	15 September 2021	[108]
PsctH (Plant Single Cell Hub)	http://jinlab.hzau.edu.cn/PsctH/ (accessed on 25 July 2024)	*Arabidopsis thaliana*, *Gossypium hirsutum*, *Oryza sativa*, *Solanum lycopersicum*, *Zea mays*	104	26 March 2024	[109]
PlantPhoneDB	https://jasonxu.shinyapps.io/PlantPhoneDB/ (accessed on 25 July 2024)	*Arabidopsis thaliana*, *Oryza sativa*, *Solanum lycopersicum*, *Zea mays*, *Populus alba × Populus glandulosa*	14,960 ligand-receptor pairs	16 July 2022	[110]
scPlantDB	https://biobigdata.nju.edu.cn/scplantdb/home (accessed on 25 July 2024)	*Arabidopsis thaliana*, *Oryza sativa*, *Zea mays*, *Gossypium hirsutum*, *Solanum lycopersicum*, *Populus alba × populus glandulosa*, *Bombax ceiba*, *Medicago truncatula*, *Triticum aestivum*, *Nicotiana attenuata*, *Manihot esculenta*, *Fragaria vesca*, *Brassica rapa*, *Catharanthus roseus*, *Gossypium bickii*, *Populus alba var pyramidalis*, *Glycine max*	229,551	10 July 2023	[111]

## Data Availability

All data are contained within the article.
